# Congenital hyperinsulinism: current trends in diagnosis and therapy

**DOI:** 10.1186/1750-1172-6-63

**Published:** 2011-10-03

**Authors:** Jean-Baptiste Arnoux, Virginie Verkarre, Cécile Saint-Martin, Françoise Montravers, Anaïs Brassier, Vassili Valayannopoulos, Francis Brunelle, Jean-Christophe Fournet, Jean-Jacques Robert, Yves Aigrain, Christine Bellanné-Chantelot, Pascale de Lonlay

**Affiliations:** 1 Centre de Référence des Maladies Héréditaires du Métabolisme de l'Enfant et l'Adulte, AP-HP Hôpital Necker-Enfants Malades, Université Paris Descartes, Paris, France; 2 Département d'anatomopathologie. AP-HP Hôpital Necker-Enfants Malades, 149 rue de Sèvres, 75743 Paris Cedex 15, Paris, France; 3 Département de Génétique, AP-HP Groupe Hospitalier Pitié-Salpétrière, Université Pierre et Marie Curie-Paris 6, Paris, France; 4 Service de Médecine Nucléaire, AP-HP Hôpital Tenon, Paris, France

**Keywords:** Congenital hyperinsulinism (HI), ^18^F-fluoro-L-DOPA positon emission tomography

## Abstract

Congenital hyperinsulinism (HI) is an inappropriate insulin secretion by the pancreatic β-cells secondary to various genetic disorders. The incidence is estimated at 1/50, 000 live births, but it may be as high as 1/2, 500 in countries with substantial consanguinity. Recurrent episodes of hyperinsulinemic hypoglycemia may expose to high risk of brain damage. Hypoglycemias are diagnosed because of seizures, a faint, or any other neurological symptom, in the neonatal period or later, usually within the first two years of life. After the neonatal period, the patient can present the typical clinical features of a hypoglycemia: pallor, sweat and tachycardia. HI is a heterogeneous disorder with two main clinically indistinguishable histopathological lesions: diffuse and focal. Atypical lesions are under characterization. Recessive *ABCC8 *mutations (encoding SUR1, subunit of a potassium channel) and, more rarely, recessive *KCNJ11 *(encoding Kir6.2, subunit of the same potassium channel) mutations, are responsible for most severe diazoxide-unresponsive HI. Focal HI, also diazoxide-unresponsive, is due to the combination of a paternally-inherited *ABCC8 *or *KCNJ11 *mutation and a paternal isodisomy of the 11p15 region, which is specific to the islets cells within the focal lesion. Genetics and ^18^F-fluoro-L-DOPA positron emission tomography (PET) help to diagnose diffuse or focal forms of HI. Hypoglycemias must be rapidly and intensively treated to prevent severe and irreversible brain damage. This includes a glucose load and/or a glucagon injection, at the time of hypoglycemia, to correct it. Then a treatment to prevent the recurrence of hypoglycemia must be set, which may include frequent and glucose-enriched feeding, diazoxide and octreotide. When medical and dietary therapies are ineffective, or when a focal HI is suspected, surgical treatment is required. Focal HI may be definitively cured when the partial pancreatectomy removes the whole lesion. By contrast, the long-term outcome of diffuse HI after subtotal pancreatectomy is characterized by a high risk of diabetes, but the time of its onset is hardly predictable.

## Definition

Congenital hyperinsulinism (HI) comprises a group of different genetic disorders with the common finding of recurrent episodes of hyperinsulinemic hypoglycemias due to an inappropriate secretion of insulin by the pancreatic β-cells [1-5]. This definition excludes insulin related hypoglycemia due to insulin resistance syndromes and to acquired hyperinsulinemic hypoglycemias (see differential diagnosis below).

The former names of HI are now obsolete: "idiopathic hypoglycemia of infancy", "nesidioblastosis", "persistent hyperinsulinemic hypoglycemia of infancy, PHHI", because HI is genetic not idiopathic, nesidioblastosis was found to be a normal feature of the pancreas in early infancy, and HI can persist from infancy to adulthood. The denomination "Congenital hyperinsulinism" should be preferred.

## Epidemiology

The estimated incidence of HI is 1/50, 000 live births (up to 1/2, 500 in Saudi Arabia because of a high rate of consanguinity). Mutations in the *ABCC8 *and *KCNJ11 *genes are the most common causes of HI and account for 40 to 45% of all cases (82% of diazoxide-unresponsive patients [6]), whereas mutations have been identified on six other genes in approximately 5 to 10% of the cases. The genetic etiology for the remaining 45-55% of patients is still unknown [7]. Fifty five to sixty percent of diazoxide-unresponsive HI are focal forms, whereas 40-45% are diffuse forms, in western countries [6].

## Clinical presentation

Hypoglycemia is the main feature of HI and gives a high risk of seizures and brain damage. Symptoms revealing hypoglycemia are various and depend on the severity of hypoglycemia and the age of the patient, ranging from asymptomatic hypoglycemia revealed by routine blood glucose monitoring to life-threatening hypoglycemic coma or status epilepticus.

***During the neonatal period***, severe hypoglycemias are revealed by seizures in half the patients. Most affected newborns are macrosomic at birth with a mean birth-weight of 3.7 kg and approximately 30% are delivered by cesarean section [8]. Hypoglycemia is permanent, both in the fasting and the post-prandial states. The mean rate of intravenous glucose administration required to maintain plasma glucose above 3 mmol/l may be as high as 17 mg/kg.min [8]. A mild hepatomegaly is frequently found. Low cortisol and growth hormone levels can be observed at the time of hypoglycemia, but these hormonal abnormalities are not diagnostic for either cortisol or GH insufficiency and they will resolve within weeks. Neonatal hypoglycemias, when severe (coma, seizures) or prolonged, expose to a poor neurological outcome. Hypoglycemias are usually diazoxide-unresponsive, except in case of perinatal stress-induced transient hyperinsulinism, syndromic HI and HI related to *HNF4A *and *GLUD1 *mutations (see below).

***During infancy and childhood***, hypoglycemias may be diagnosed between one and twelve months of age, in half the patients, or even later in life, sometimes because of a delayed diagnosis. The presenting symptoms before 1 year of age are seizures, episodes of drowsiness or excitability. After 1 year, the symptoms are typical of hypoglycemia: pallor, faint, tachycardia and sweating, seizures. Macrosomia at birth is often reported (mean birth-weight around 3.6 kg) [8]. The characteristics of hypoglycemia are similar, although lower rates of intravenous glucose are required to maintain normal plasma glucose levels (12-13 mg/kg.min when the patient is less than 1 year old at diagnosis).

***Syndromic HI ***are usually diazoxide-responsive. The onset of hypoglycemia is usually early at birth, at a time the dysmorphic features may be barely apparent, so that careful examination and systematic diagnostic tests should be performed (e.g. CDG syndrome). Conversely, asymptomatic hypoglycemia may also be screened during the follow-up of a known syndromic patient. The specific clinical features of these syndromic HI are listed in Table 1.

**Table 1:** Syndromic HI, main clinical and genetics features

Syndrome	Inheritance	Gene	DD	LGA	**Sk.M**.	Syndact	HH	HD&M	IM	FAC	LQT	CL	Tumors	CCA	CA	Deaf	RP
**BWS**	AD or S	11p15.5		X			X						X				
**Perlmann**	AR	?		X				X	X				X	X			
**SGB**	XL	Glypican3	X	X	X			X	X				X	X	X	X	
**CDG-Ia**	AR	PMM2	X					X							X		X
**CDG-Ib**	AR	PMI															
**Kabuki**	AD or S	MLL2	X		X			X	X								
**Sotos**	S	NSD1	X	X	X			X					X	X	X	X	
**Timothy**	AD or S	CACNA1C	X			X		X			X						
**Costello**	AD or S	HRAS	X					X				X	X				
**Ondine**	AD or AR	PHOX2B											X				
**Usher Ic**	AR	USH1C								X						X	X

The "X" indicates when the symptom can be present. **AD**: Autosomal Dominant; **AR**: Autosomal recessive; **BWS**: Beckwith-Wiedemann syndrome; **CA**: Cerebellar Atrophy or Hypoplasia; **CCA**: Corpus Callosum Agenesia; **CDG**: Congenital Disorder of Glycosylation; **CL**: Cutis Laxa; **DD**: Developmental Delay; Deaf: Deafness; **FAC**: Failure of Autonomic Control; **HD&M**: Heart Defect or Malformation; **HH**: Hemi Hypertrophy; **IM**: Intestine Malformation (Volvulus, ileal atresia, Meckel's diverticulum, intestinal malrotation); **LGA**: Large for Gestational Age; **LQT**: Long QT syndrome; **RP**: Retinitis Pigmentosa; **SGB**: Simpson-Golabi-Behmel syndrome; **Sk. M**: Skeletal Malformation; **S**: Sporadic; **Syndact**: syndactyly; **Tumors**: higher risk for tumors; **XL**: × linked; **11p15.5**: Imprinting abnormality or paternal disomy of the 11p15.5 chromosomal region.

## Etiopathogenesis

Congenital hyperinsulinism is a primary defect of the pancreatic β-cell leading to an inappropriate secretion of insulin [5]. Insulin lowers plasma glucose both by inhibiting glycogenolysis and gluconeogenesis and by stimulating glucose uptake in muscle and adipocytes. This explains two main and characteristic clinical findings of neonatal HI: the high glucose infusion rate required to prevent hypoglycemia and the responsiveness of hypoglycemia to exogenous glucagon, since glucagon stimulates glycogen breakdown and gluconeogenesis. Moreover, insulin inhibits lipolysis, thus preventing compensatory ketogenesis to protect the brain from hypoglycemia.

## Classification of isolated HI

### 1. Pathological classification

We present here a classification according to the etiopathogenic process.

***Channelopathies ***affect the subunits of a K_ATP _channel set through the plasma membrane of the β-cells. Both subunits can be affected: the sulfonylurea receptor (SUR1) encoded by the ***ABCC8 ***gene and the inward-rectifying potassium channel (Kir6.2) encoded by the ***KCNJ11 ***gene. When closed, the K_ATP _channel depolarizes the plasma membrane leading to insulin secretion. As diazoxide is a K_ATP _channel agonist, the patients are diazoxide-unresponsive when the defect in *ABCC8 *or *KCNJ11 *abolishes the function of this channel. In this group of HI, two clinically indistinguishable histopathological lesions have been described: the focal and the diffuse HI (both mostly resistant to diazoxide). Focal HI is sporadic, while diffuse HI is autosomal-recessively inherited [9-15] or more rarely dominantly-inherited [16].

***Enzymes anomalies ***or other metabolic defects involve glucokinase encoded by the **GCK **gene [17], glutamate dehydrogenase or GDH encoded by ***GLUD1 ***gene (HI/HA syndrome) [18], short-chain L-3-hydroxyacyl-CoA dehydrogenase (SCHAD) encoded by ***HADH ***gene [19], and more recently the ***SLC16A1 ***gene encoding a monocarboxylate transporter (MCT1) that mediates the movement of lactate and pyruvate across cell membranes and causes physical exercise-induced hypoglycemia (the patients suffer from hypoglycemic symptoms only when performing strenuous physical exercise) [20] and the ***UCP2 ***gene encoding the UCP2 protein which regulates the protons leak across the inner mitochondrial membrane [21]. *UCP2 *(mitochondrial uncoupling protein 2) mutations were recently reported in two unrelated children with neonatal-onset congenital HI and hypoglycemia which were diazoxide-responsive [21]. UCP2 induces a regulated leak of protons across the inner mitochondrial membrane and uncouples mitochondrial oxidative metabolism from ATP synthesis [22]. Consequently, ATP cells content decreases and insulin secretion as well. UCP2 over-expression in rat isolated pancreatic islet cells decreases the ATP content and inhibits glucose-stimulated insulin secretion. Conversely, UCP2 knockout mice exhibit hyperinsulinemic hypoglycemia [23].

All these types of HI affect the ATP/ADP ratio within the β-cells, which determines the opened or the closed status of the K_ATP _channel. They are diffuse forms of HI and diazoxide-responsive with the exception of GCK mutations which can be diazoxide-unresponsive in some patients.

***A transcription factor ***defect, involving the hepatocyte nuclear factor 4 alpha gene (***HNF4A***), is also responsible of HI. The target genes of HNF4A are various and not fully explored. HNF4A may promote Kir6.2 expression [24], have an interaction with the peroxysome proliferators-activated receptor- α (PPAR-α) which has a known role on cellular lipid metabolism. PPAR-α null mice exhibit a fasting hyperinsulinemic hypoglycemia phenotype [25]. This dominantly-inherited HI, occurring at birth, is diazoxide-responsive and has a diffuse pattern at the histopathological examination. Relatives may present MODY 1 type diabetes.

### 2. Histological classification

**Figure 1:**
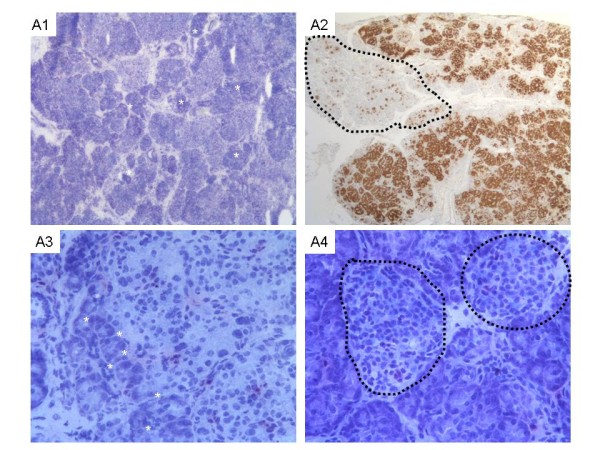
**Histology of the focal form of HI**. **Histological features of focal form **(A1-A4) on frozen sections stained by toluidine blue (A1, A3, A4) and on immunostaining with proinsuline antibody (A2). At low magnification (A1, x25; A2, x16) a modified architecture of pancreatic tissue is observed within the focal form. The focal form is not encapsulated and is poorly delimited. It contains focal adenomatous hyperplasia of islets (pale areas) intermingled and/or surrounded by exocrine acini (darkest area underlined by white stars) (A1 and A3). This pattern is underlined by proinsuline showing an evident contrast between the focal lesion and normal pancreas (doted line circles). At high magnification (A3 and A4, × 200), the focal lesion (A3) is composed of islets containing a heterogeneous population of endocrine cells of various sizes. Some of these cells have large nuclei (arrows, A3) and large cytoplasms. By contrast, normal islets (doted line circles) observed outside the lesion have endocrine cells of usual size without enlarged nuclei (A4).

***Focal forms ***are usually restricted to a small area (2.5 to 7.5 mm in diameter) of the pancreas but very large focal forms were also observed. The focal lesions are made of large endocrine cells because of their large cytoplasm with dispersed abnormal nuclei of irregular and angular shape that are more than 3 to 5 times the size of nearby acinar nuclei taken as controls (Figure 1). Most of the time, a focal lesion contains multiple adjacent abnormal lobules frequently intermingled with or surrounded by acinar foci. Somatostatin detection by immunohistochemistry reveals a second endocrine-cell population within the focal lesion which is not exclusively composed of β-cells. The focus is poorly delimited, but an organoid pattern remains, suggesting an abnormal developmental process more than a tumor as it does not invade or push the margins, and there is no pseudocapsule. The area of abnormal pancreatic development is multilobular and can have satellites in the nearby pancreas that necessitate intraoperative margins analysis to ensure complete excision and avoid recurrence [26-28].

**Figure 2:**
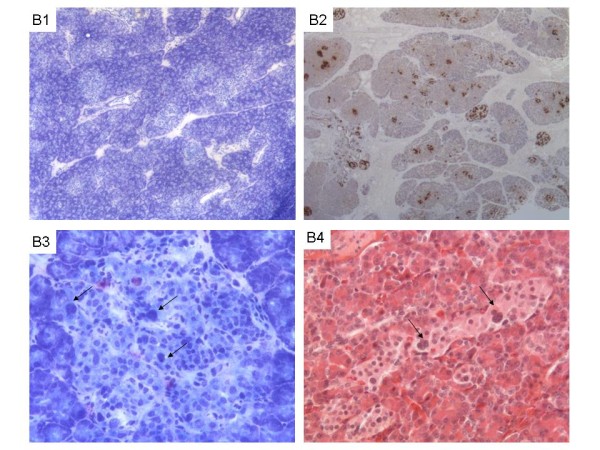
**Histology of the diffuse form of HI**. **Histological features of diffuse form **(B1- B4) on frozen sections stained by toluidine blue (B1, B3) or on formalin fixed paraffin embedded section stained with HES (B4) or with proinsuline antibody (B2). At low magnification (B1, x25; B2, x16), the architecture is preserved with a normal distribution between exocrine (dark) and endocrine area (pale). At higher magnification (B3 and B4 x200), abnormal endocrine islets contain cells with voluminous nuclei (arrows, B3) and enlarged cytoplasm. The entire pancreas is interspersed with abnormal islets intermingled with morphologically normal islets in a variable proportion (B4). To confirm a diffuse form on extemporaneous frozen sections, abnormal islets must be observed on biopsies sampled from at least 2 or 3 distinct areas of the pancreas.

***Diffuse form ***means a disease involving every β-cell throughout the pancreas with a variable involvement of the islets. The islet pattern is preserved, but it contains very active β-cells with very abundant cytoplasm and highly abnormal nuclei 3 to 4 times the size of acinar nuclei (Figure 2). These abnormalities vary in intensity from one islet to another [26-28].

***Atypical forms ***are not very well defined. The pattern of pancreatic involvement is of the diffuse type but it is found only in one large area of the pancreas [29]. Their genetic basis is not yet totally elucidated but some are caused by chromosomal mosaics.

### 3. Genetic classification

Two situations should be considered:

***Focal islet-cell hyperplasia ***is associated with homozygosity of a paternally inherited mutation of *ABCC8 *or *KCNJ11 gene*s in the hyperplastic pancreatic islets, which is due to a heterozygous paternally inherited mutation and a loss of the maternal allele with a paternal isodisomy at the same locus [30-32]. Both *ABCC8 *and *KCNJ11 *genes are on chromosome 11p15.1.

***Diffuse hyperinsulinism ***is heterogeneous in the involved gene and the mechanism of inheritance. Table 2 summarizes the genes, modes of inheritance and histological forms of isolated HI.

**Table 2:** Summary of genetic causes of isolated HI

	Gene	Protein	Inheritance	**Diazoxide-Resp**.	Histology	Comment
**K**_**ATP **_**Channel**	ABCC8	SUR1	AR	No	F or D	
			AD	Usually	D	
	KCNJ11	Kir6.2	AR	No	F or D	
**Enzymes/Transporters**	GLUD1	GDH	AD or DN	Yes	D	HIHA syndrome
	GCK	GCK	AD or DN	Usually	D	MODY 2
	HADH	SCHAD	AR	Yes	D	
	SLC16A1	MCT1	AD	Usually	D	EIHI
	UCP2	UCP2	AD	Yes	D	
**Transcription Factor**	HNF4A	HNF4A	AD or DN	Yes	D	MODY 1

**AR**: autosomal recessive; **AD**: autosomal dominant; **DN**: De Novo; **F**: Focal Form; **D**: Diffuse Form; **HI/HA**: hyperammonemia/hyperinsulinism syndrome; **EIHI**: Exercise-induced hyperinsulinism; **GDH**: Glutamate Dehydrgenase; **GCK**: Glucokinase; **HADH**: Hydroxy-Acyl-CoA Dehydrogenase; **MCT1**: Monocarboxylate transporter 1; **MODY**: Maturity-onset diabetes of the young: **UCP2**: Uncoupling protein 2.

#### 3a. Antenatal diagnosis

Antenatal diagnosis can be proposed in case of severe diffuse isolated HI, when mutations are identified in a proband. It will follow the same procedure as for other genetic diseases with a molecular analysis on a chorionic villies biopsy or the amniotic fluid. The clinical phenotype of severe diffuse HI is homogeneous in a family (*ABCC8 *or *KCNJ11*). However, *GLUD1 *or *HNF4A *phenotypes may present major differences among patients of a same family concerning the neurological pattern and the occurrence of MODY, respectively. We don't consider relevant antenatal diagnosis for diazoxide-responsive isolated HI, since an appropriate management at birth will allow quick diagnosis and avoid severe hypoglycemia, followed by an easy and safe subsequent treatment with diazoxide.

#### 3b. Genetic counselling

Four situations must be considered:

- De novo mutation: the risk of recurrence is null.

- Focal form: the risk of recurrence among the siblings is almost negligible and was observed only once [33]. The loss of heterozygosity of the paternal mutation is a rare sporadic event, as suggested by discordant identical twins and absence of HI in the patients' fathers. In case of consanguineous parents, the paternal mutation responsible for a focal form in a family has to be screened in the mother for the next pregnancy, to exclude the possibility of a diffuse HI by a homozygous mutation, inherited by each parent [34].

- Diffuse form: the genetic counseling will depend on the mode of inheritance. The risk of recurrence is 25% for each new pregnancy when the disease is recessively inherited, whereas this risk reaches 50% when the disease is dominantly inherited.

- When the genetic testing was not able to identify the causative gene, a theoretical risk for recurrence for siblings exists, 25 or 50% according to the suspected inheritance mode. Siblings must be screened for hypoglycemia during the first few days of life to avoid hypoglycemia related brain damage.

## Diagnosis

The diagnostic criteria for HI include:

- Fasting and/or post-prandial hypoketotic hypoglycemia (< 2.5 - 3 mmol/l)

- Inappropriate plasma insulin levels (plasma insulin concentration detectable) and c-peptide concomitant to hypoglycemia. Indeed, insulin levels should be undetectable at the time of hypoglycemia. In HI patients, plasma insulin levels are not frequently high during hypoglycemia, and they remain within the normal range of the laboratory. However, normal ranges of insulin levels were set with normal blood glucose.

- An increase in blood glucose greater than 1.7 mmol/L (30 mg/dL) within 30 - 40 minutes after IM or IV administration of 1 mg glucagon. Indeed insulin promotes glycogen storage and inhibits its use. Conversely, glucagon stimulates glycogenolysis. When glucagon increases blood glucose, it proves the paradoxical hepatic glycogen content despite hypoglycemia, and it rules out the differential diagnosis of glycogen storage disease.

- Inappropriately low ketone bodies in plasma and urines and low free fatty acids in plasma even for fasting hypoglycemia (Insulin inhibits lipolysis) [35].

A major specific but inconstant diagnostic criterion is the glucose infusion rate required to maintain blood glucose above 3 mmol/L. A glucose infusion rate higher than 10 mg/kg.min in a neonate proves an insulin related hypoglycemia. This threshold decreases with age: 7 mg/kg.min in 5 years old children and 4 mg/kg.min in adults. Indeed, most hormones (cortisol, growth hormone, glucagon, epinephrine etc.) and metabolic pathways sustain blood glucose by stimulating hepatic glycogenolysis and gluconeogenesis leading to a physiological glucose output up to 10 mg/kg.min in neonates. Thus, if hypoglycemias are still occurring despite higher glucose infusion rate, the only possible mechanism is insulin. Protein-induced hypoglycemia can be observed in HI, especially in HI/HA syndrome (see HI/HA syndrome below).

Once the diagnosis of hyperinsulinemic hypoglycemias is set, further routine evaluations are necessary to precise the etiology. Indeed, patients may present an "**isolated HI**" secondary to a primary disorder of insulin secretion or a "**syndromic HI**" where HI is one symptom in the phenotype of a more generalized disease. Most patients have isolated HI with no other associated symptoms but a subtle facial dysmorphism [36]. Some of these etiologies are screened with routine tests:

- Ammonemia: hyperammonemia may lead to the diagnosis of HI/HA syndrome,

- Urine organic acids (high 3-OH-glutarate) and plasma acylcarnitines (high C_4_-OH-carnitine), to identify a short-chain-hydroxyacyl-CoA dehydrogenase (SCHAD) deficiency.

- Clinical examination searching for dysmorphic features (hemihypertrophy, overgrowth, fat pads, congenital heart defects etc.) may require further biochemical, imaging or genetics tests to confirm the diagnosis of syndromic HI.

Syndromic HI includes (Table 1):

- Congenital Disorder of Glycosylation syndrome type Ia and Ib [37]

- Kabuki syndrome [38]

- Costello syndrome [39]

- Thimoty syndrome [40]

- Usher syndrome type Ic [41]

- Ondine syndrome [42,43]

- Overgrowth syndromes

○ Beckwith-Wiedemann syndrome [44-46]

○ Perlman syndrome [47]

○ Simpson-Golabi syndrome

○ Sotos syndrome [48]

In the following chapters, only the management and the genetics of isolated HI will be reviewed. The management of syndromic HI won't be specifically described, because they are diffuse, usually diazoxide-responsive and require extra care for their supplementary symptoms.

## Differential diagnosis

### Perinatal-stress induced HI

This occurs under specific conditions and the pathophysiology remains unclear. It appears to be acquired and not genetic. It lasts from several hours to 2 to 3 months and may require transient diazoxide therapy. This entity includes hyperinsulinemic hypoglycemia in newborns from mother with unstable diabetes, small fetus for gestational age, neonates with fetal distress, birth asphyxia etc.

### Drug-induced hyperinsulinemic hypoglycemia

- Oral antidiabetic drug (sulfonylurea, glinides, biguanide, gliptine).

- Beta Blockers.

- Numerous other drugs: antiarrhythmic drugs (cibenzoline, disopyrapide, quinine, flécaine), LHRH analogue, conversion enzyme inhibitor, antiviral drugs, leukocytes growth factors, Interferon, Triptan etc.

- Munchhausen syndrome and Munchhausen by proxy syndrome by insulin injection (high plasma insulin, low c-peptide at the time of hypoglycemia) or by sulfonylurea administration (high insulinemia, high c-peptide and presence of sulfonylurea in plasma and urine at the time of hypoglycemia) [49].

### Insulinoma

Insulinoma has an incidence of about 1/1, 000, 000 in adults and is exceptional in children. In a recent review, the youngest patient was 8 years old and the median age at presentation was 47 years [50] or 25 years in case of MEN-1 [51]. Thus, when hypoglycemia occurs during childhood, an insulinoma must be considered.

### Insulin resistance syndrome (IRS)

IRS is a defect of the target cells of insulin [11]. It leads mostly to hyperinsulinemic hyperglycemia but also to hyperinsulinemic fasting hypoglycemia. Two types of IRS are described, depending on whether the cause is genetics (type A: mutation in the insulin receptor gene or in the post-receptor metabolic pathways) or auto-immune (type B: anti-insulin receptor antibodies).

### Proinsulinemic hypoglycemia

This exceptional condition with post prandial hypoglycemias was described in patients with defects in the processing of prohormones (*PCSK1 *gene). Patients may present with hyperphagia, chronic diarrhea, severe obesity, hypocortisolism and hypogonadotropic hypogonadism [52].

### Hyperinsulin-like hypoglycemia

Non Islet cells tumor hypoglycemia (NICTH) is due to an oversecretion of incompletely processed precursors of IGF-2 ('big'-IGF-2) by the tumor. It mainly occurs in solid tumors of mesenchymal and epithelial origin, but also rarely in hematopoietic and neuroendocrine tumors [53,54].

### Hypoglycemia not related to inappropriate insulin secretion or clearance

This category includes various endocrine and inherited metabolic diseases.

## Management

Rapid diagnosis and prompt management of hypoglycemias are vital to prevent brain damage and intellectual disability [55]. The severity of HI is evaluated by the rate of glucose infusion required to maintain normoglycemia and the response to medical treatments.

### Drugs

Three drugs are currently used in the treatment of HI.

*- Glucagon *is a polypeptide hormone consisting in 29 amino acids. It is physiologically secreted by the α-cells of the pancreatic islets to promote glycogenolysis [20]. Glucagon is essential as an emergency treatment of severe hypoglycemia but it is not appropriate as a long-term treatment.

*- Diazoxide *is an antihypertensive antidiuretic benzothiadiazine. Its action on the pancreatic β-cells opens the K_ATP _channel, thereby inhibiting insulin secretion [56]. Oral diazoxide is used at the initial dose of 5-15 mg/kg.day (neonates) or 10 mg/kg.day b.i.d or t.i.d [57]. Later dose adjustments may be necessary, based on its ability to maintain euglycemia. Tolerance to diazoxide is usually good. The most frequent adverse effect is hypertrichosis, which can sometimes be marked and distressing in young children, but will be reversible after this treatment disruption. Hematological side effects are very rare with the usual doses. Severe adverse effects were observed: sodium and fluid retention may precipitate congestive heart failure in patients with compromised cardiac reserve, but usually responds to diuretic therapy. Moreover, life-threatening episodes of pulmonary hypertension were observed in some neonates receiving diazoxide [58]. These heart and vascular complications are mostly observed in preterm children, raising the question of its contraindication in premature patients.

*- Somatostatin analogues*. Octreotide is proposed in case of non-responsiveness to diazoxide [59]. It is administered SC (injections/6 to 8 hours or continuously with a pump) or IV because of its short half life (1 to 2 hours). Tachyphylaxis may limit the efficiency of octreotide. It leads to a rapid decrease in the response to octreotide 24-48 hours after initiation of this treatment. As a consequence, the response to octreotide can be assessed only 2 days after the initiation of a new daily dose. In case of unresponsiveness, a higher dose may be tried. The maximal dose ranges between 15 and 50 μg/kg.d according to the HI referent centers. Most side effects occur just at initiation of the treatment: vomiting and/or diarrhea and abdominal distension, that resolve spontaneously within 7-10 days. However, fatal necrotizing enterocolitis has also been reported in some neonates [60]. Gallbladder sludge or stones are rare long term complications which should be screened with abdominal ultrasounds. Ongoing studies on long-acting somatostatin analogues investigate their effectiveness and their impact on the patients' quality of life, in different HI referent centers in the world.

### Medical management (Figure 3 and 4)

In neonates, the treatment must be diligently and intensively performed to prevent irreversible brain damage [55]. This chapter will focus on the management of severely hypoglycemic neonates but the protocol should be lightened for less severe patients.

**Figure 3:**
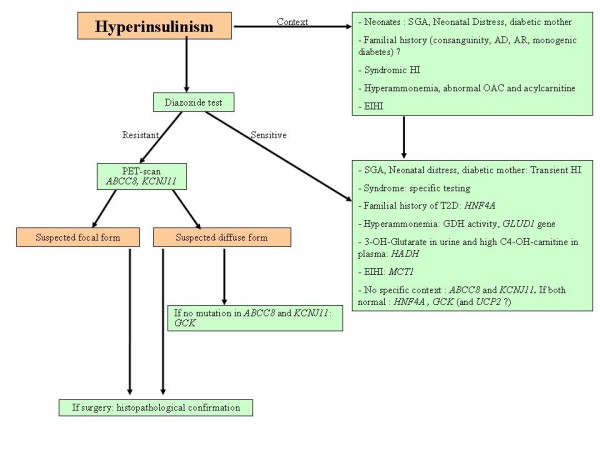
**Diagnostic tree for HI**. AD: Autosomal Dominant; AR: Autosomal Recessive; MODY: Maturity Onset Diabetes of the Youth; HI: Hyperinsulinism; OAC: Organic Acid Chromatography; EIHI: Exercise-Induced HyperInsulinism; SGA: Small for gestational age. T2D: Type 2 diabetes.

**Figure 4:**
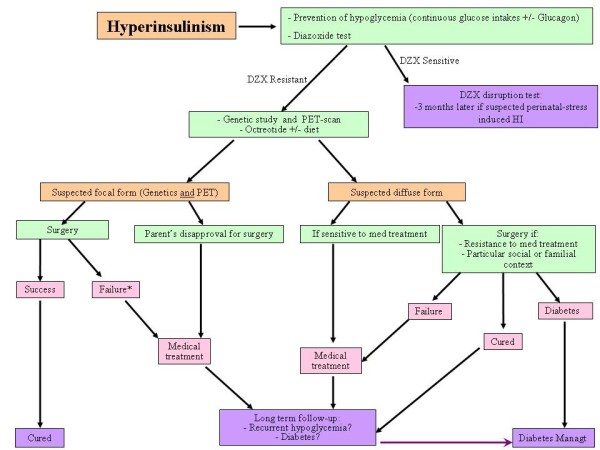
**Management tree for HI patients**. DZX: Diazoxide; HI: Hyperinsulinism; Med treatt: Medical treatment. * Failure of the surgery for a focal form of HI is rare, but happens when the focal form is very large or when two focal forms coexist within the same pancreas.

#### Stabilization

In case of severe hypoglycemia, 1 mg Glucagon is used IM, IV or SC, until a (central) IV line is set for continuous glucose infusion. Indeed, the need for glucose may exceed the gastrointestinal tolerance of neonates, so that continuous IV glucose infusion will be associated to continuous feeding [61]. It is important to determine the minimum glucose infusion rate required to maintain normoglycemia both for diagnosis purpose (a rate > 10 mg/kg.min is a specific diagnostic criteria for HI) and to limit fluid infusion/retention. Continuous IV glucagon (1 to 2 mg per day) can be added when blood glucose remains unstable despite a high glucose infusion rate. The severity of hypoglycemia or the rate of glucose required to maintain normoglycemia cannot foretell the form of HI: transient, genetic, focal or diffuse.

#### Treatment initiation

When the diagnosis is set without any spontaneous improvement within the first few days of life (most transient HI: e.g. gestational diabetes), a specific treatment of HI must be initiated. Diazoxide is started for a 5 days trial. Diazoxide responsiveness criteria is the absence of hypoglycemia (> 3 - 3, 8 mmol/L) under a normal diet and during a 8-12 hr fast. If diazoxide does not meet these criteria, octreotide is added and the patient is screened for focal forms of HI (genetic testing and PET scan). Octreotide is started at an initial dose of 5-10 μg/kg.d, and its response will be assessed 48 hours later. When unresponsive, the dose will be slowly titrated up to 15 - 50 μg/kg.d depending on the standard of care of the HI reference center. The criteria for a fully responsive patient to octreotide are the same as for diazoxide.

#### In case of response to medical therapy

Patients can experience spontaneous clinical improvements that occur relatively early (within several months) or later (several years). Medications can be disrupted during childhood for most patients; however some may still require diazoxide or octreotide for decades. The need for a long term therapy does not apply to perinatal-stress induced HI.

#### In case of unresponsive or partially responsive HI

Glucose must be provided to maintain normoglycemia. This may require frequent glucose enriched oral feedings, frequent or continuous enteral feedings and continuous IV glucose infusion. Diazoxide and/or octreotide can be continued when a partial response was observed.

As patients with severe HI can experience spontaneous, but partial improvement within the first months of life, the surgical indication must be carefully discussed.

#### Diagnosis of focal vs. diffuse HI (PET-scan and Genetics)

Precise genetic and PET-scan diagnosis are essential in the management of diazoxide-unresponsive patients [62-64], because:

- Close to 50% of diazoxide-unresponsive patients have focal forms for which limited surgery may lead to total healing with minor risk of complications [64,65].

- Some severe HI patients remain unstable with persistent hypoglycemia despite an intensive medical treatment. Subtotal pancreatectomy can be considered to reduce the risk of brain damage due to recurrent hypoglycemia.

- Genetics and PET-scan provide informations:

○ for the diagnosis of focal or diffuse form of HI, thus participating in the indication of surgery,

○ to determine the type of surgery: partial pancreatectomy in case of a focal form or subtotal pancreatectomy for diffuse HI [66-70].

#### ^18^F-fluoro-L-DOPA positron emission tomography (PET)

**Figure 5:**
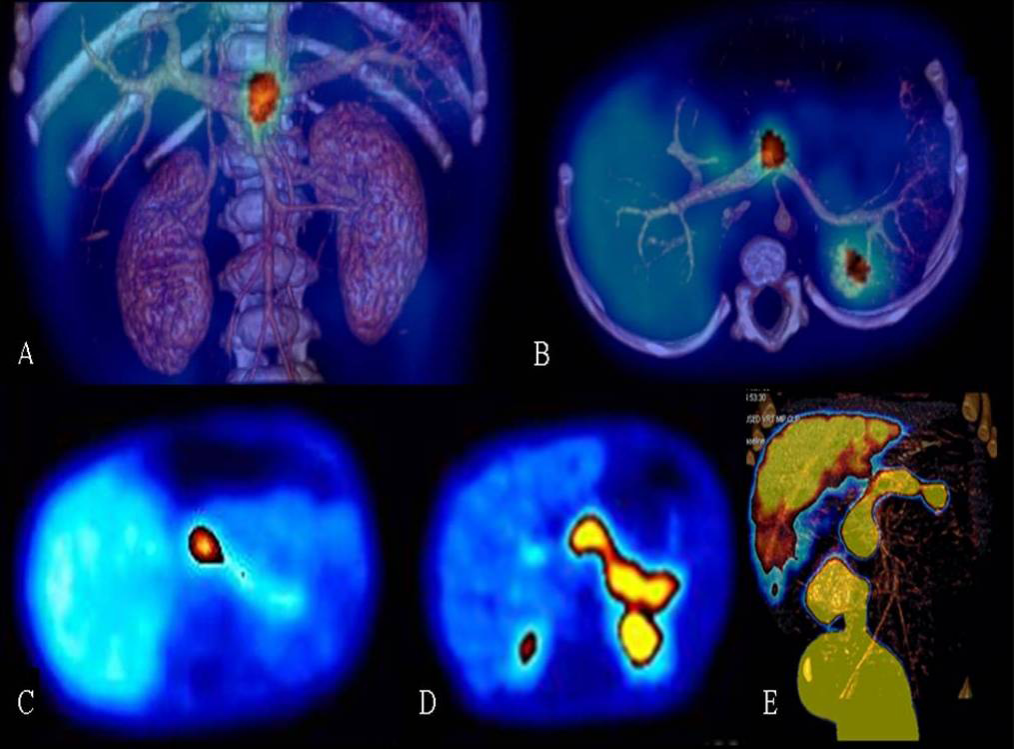
**Abdominal ^18^F-fluoro-L-DOPA PET-scan imaging in HI**. Focal form (A, B, C): PET-scan localizes accurately the focal lesion. A: Anterior view of a 3 dimensions CT-scan reconstruction fused with PET imaging. B: Transversal view (Fusion PET&CT) of the same patient with a focal form. The pancreatic uptake of the radiotracer is almost exclusively located at the head of the pancreas with a near-complete silencing of the rest of the pancreatic tissue (C: Transversal PET imaging). In a suspected diffuse form, the uptake of the radiotracer appears in the whole pancreas (D: PET, transversal view; E: Pet and 3D CT fusion). Fluoro-DOPA is excreted in the kidneys and the bile, so that liver (C, D, E), kidneys (D, E), ureteras, bladder and diaper (E) appear on the PET imaging.

^18^F-fluoro-L-DOPA PET diagnoses 75% of focal cases and is 100% accurate in identifying the location of the lesion [28,71-76]. The precision seems better when the PET imaging is fused with a CT-angiography (Figure 5). A 4 hr fast is recommended prior to the PET study to avoid interference with bile secretion, as fluoro-DOPA is excreted through the kidneys and in the bile.

#### Genetics (see section above)

The diagnosis of diffuse form is definitive when two mutations are found in *ABCC8 *or *KCNJ11 *genes, or mutation(s) in the other genes involved in HI. In that case, PET-scan is not necessary.

A focal form is suspected only when one paternally inherited mutation is discovered in the *ABCC8 *or *KCNJ11 *gene. However, genetics alone may not be diagnostic for the focal form since i) a second mutation may have been missed, and ii) a recent publication reports several diffuse HI patients (histological confirmation) carrying a single heterozygous mutation, mostly paternally-inherited [6]. Thus, in patients carrying a single K_ATP _channel mutation, genetic analysis must be confronted with the PET imaging to categorize the form of HI, diffuse or focal.

#### Surgical treatment

Surgery is recommended in case of a focal form but is required when medical and dietary therapies are ineffective to maintain normoglycemia. The indication for surgery in case of severe diffuse HI may vary according to the experience of each center. It is recommended to stop all medications before the intervention (5 days for diazoxide, 2 days for octreotide) as these drugs may interfere with the histological analysis. The first step of the intervention is to confirm the diagnosis of focal or diffuse form of HI by performing biopsies of the head, body and tail of the pancreas.

Once the confirmation is given:

- In case of a focal form (normal biopsies, unless one sampled directly from the suspected focal form), the lesion will be excised. Per operative histology will search for abnormal cells at the margins. If so, additional resections will be performed until the margins are clear. The patient will be totally cured without major complication. Persistent hypoglycemia immediately after surgery may account for an incomplete resection or an exceptional distinct second focal form in the same patient. In our series of 15 patients operated for a focal form over the last three years, 2 patients required a second surgery either because of persistent abnormal β-cells at the margins of a very large focal form (1 patient), or because of a co-existing and distinct 2^nd ^or 3^rd ^focal form in the same patient (1 patient). Despite the 2^nd ^intervention, these two patients have still hypoglycemias and thus continue an intensive medical treatment.

- Diffuse form: histology shows abnormal β-cell nuclei in all sections of the pancreas. Diffuse HI requires near total pancreatectomy (95 - 98% of the pancreas) leaving just the small triangle of pancreatic tissue spanning from the duodenum to the common bile duct. The immediate post surgical outcome is unpredictable: disturbance in glucose homeostasis still remains (persistent hypoglycemia in about 50% of patient, insulin-requiring diabetes in 20% of patients during the post-surgical period) but usually in a more manageable manner than before surgery. Pancreatic exocrine insufficiency will be treated with pancreatic enzymes.

#### Particular case: HI/HA syndrome

Patients with HI/HA syndrome requires a long term treatment with diazoxide. However, a restricted protein diet, limiting the leucine intake to 200 mg per meal, is possible as an adjuvant treatment.

## Long-term outcome

**Figure 6:**
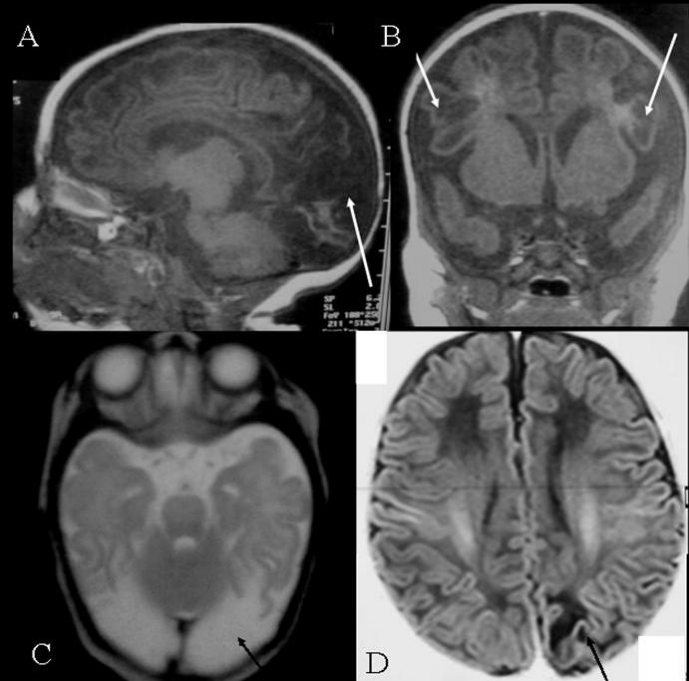
**Cerebral RMI: Brain damage after severe hypoglycemia in HI**. A, B, C: Brain RMI of a patient with severe necrotic lesions of the occipital lobes, but also cysts of parietal lobes. D (Flair sequence): Another patient presenting with a characteristic unilateral occipital lesion.

Severe brain damage is the consequence of deep and prolonged hypoglycemias presenting as coma and/or status epilepticus in neonates (Figure 6, Brain RMI: Typical lesion of severe hypoglycemia). In older children, hypoglycemias are usually less severe and brain damage is less frequent. Psychomotor skills and neurological disabilities were studied in a series of 90 HI patients (among them 63 had surgery). An intellectual disability was observed in 26% of the patients; the deficit was severe in 8% of them.

Major intellectual disability was more frequent:

- in neonatal-onset patients: 11% vs. 3% when HI was diagnosed later in infancy.

- in patients who underwent surgery: 10% vs. 4% when HI required no surgery. Within the operated group, there was no difference between patients with focal and diffuse forms.

In patients with moderate disability, there was no difference according to the age at onset of symptoms, the medical or surgical treatment or the type of histological lesion [77-79].

Patients with HI/HA syndrome have a different outcome. Indeed, half the patients with HI/HA syndrome have a progressive intellectual deficit and/or epilepsy unrelated to hypoglycemia. Thus, follow-up should include EEG with photopic stimulation and evaluations of motor and intellectual performances [80].

Glucose intolerance and diabetes are also long term complications of HI, especially in patients with a mutation in *HNF4A *gene or those who underwent a near-total pancreatectomy. In the latter, an insulin therapy for diabetes was started in 91% of the patients within 14 years after surgery (personal data). Thus, their follow-up must include a regular assessment of glucose tolerance as diabetes can appear only several years after surgery.

## Perspectives for future

The management of severe HI patients is challenging and requires a multi disciplinary team: clinicians, physicians, surgeons, pathologists, geneticists and basic scientists. Three main themes are the focus of research:

1) to improve the medical treatment of HI when not cured by surgery. Some new treatments are under evaluation (exendin (9-39) [81], long acting somatostatin analogues). Calcium-channel blockers, like nifedipine, showed a clear efficiency in mouse models [82] and in isolated case reports [83,84], but most HI centers, including ours, never observed any response to nifedipine in large series of patients. Its use should be further evaluated to determine whether some specific indications can be retained.

2) to improve the accuracy of the diagnosis and of the characteristics (localization and size) of focal forms. DOPA-PET is very accurate in locating the focal form, however for many patients, it may lack in sensitivity. The use of new radiotracers, more specific to the hypersecreting β-cells may improve the accuracy and the sensitivity of this preoperative test. In the operation theater, the challenge is to better locate the focal lesion and to perform the most selective surgery. Peroperative tests, such as pancreatic ultrasonography, are under evaluation [85]

3) to search for new genes involved in HI: About 50% of diazoxide-responsive and 20% of diazoxide-unreponsive patients have no genetic explanation for their disease. Families of patients are actually being studied to bring light on the mechanisms of HI. Candidate genes are various and may involve genes such as glucose transporters, enzymes involved in energy metabolism (glycogenolysis or mitochondrial modulators), transcription factors, ion channels etc.

At last, treatment strategies differ greatly among teams worldwide because of the lack of data about the long term neurological and glycemic outcome of patients according to the type of management (surgery or intensive medical treatment). There are also no precise data about the clinical remission some patients can experience and its possible mechanisms (adaptation of the energy metabolism of the β-cells or of counter-regulation hormones, premature apoptosis of the hyper-secreting β-cells ...). Further collaborative studies and an international database may certainly help for a better understanding of the disease and to set up an international therapeutic consensus.

## List of abbreviations used

**ATP/ADP: **adenosine tri-phosphate/adenosine bi-phosphate; **b.i.d: **"bis in die" meaning "twice a day"; **CDG: **Congenital disorder of glycosylation; **EEG: **Electroencephalogram; **EIHI: **Exercise-induced Hyperinsulinism; **IM: **intra muscular; **IV: **intra venous; **MEN: **multiple endocrine neoplasia; **RMI: **Resonance magnetic imaging; **SC: **subcutaneous; **t.i.d: **"ter in die" meaning "three times a day".

## Competing interests

The authors declare that they have no competing interests.

## Authors' contributions

JBA and PDL wrote and coordinate the writing of the manuscript; VVe and JCF performed the histological figures; CSM and CBC wrote the genetics section; YA supervised the writing of the surgical management section; FM created the PET-scan figure; AB, VVa, FB and JJR participated in the writing of the medical management section; JJR participated in the writing of the long-term outcome section; FB created the brain RMI figure. All the authors read and approved the final manuscript.
